# Engineering and crystal structure of a monomeric FLT3 ligand variant

**DOI:** 10.1107/S2053230X21003289

**Published:** 2021-04-06

**Authors:** Erwin Pannecoucke, Laurens Raes, Savvas N. Savvides

**Affiliations:** a Unit for Structural Biology, Department of Biochemistry and Microbiology, Ghent University, Technologiepark-Zwijnaarde 71, 9052 Zwijnaarde, Belgium; b Unit for Structural Biology, VIB Center for Inflammation Research, Technologiepark-Zwijnaarde 71, 9052 Zwijnaarde, Belgium; c Laboratory for General Biochemistry and Physical Pharmacy, Ghent University, Ottergemsesteenweg 460, 9000 Gent, Belgium

**Keywords:** FLT3 ligand, FLT3, receptor tyrosine kinases, acute myeloid leukemia

## Abstract

The overarching paradigm for the activation of class III and V receptor tyrosine kinases (RTKs) prescribes cytokine-mediated dimerization of the receptor ectodomains and homotypic receptor–receptor interactions. However, structural studies have shown that the hematopoietic receptor FLT3, a class III RTK, does not appear to engage in such receptor–receptor contacts, despite its efficient dimerization by dimeric FLT3 ligand (FL). As part of efforts to better understand the intricacies of FLT3 activation, we sought to engineer a monomeric FL. It was found that a Leu27Asp substitution at the dimer interface of the cytokine led to a stable monomeric cytokine (FL_L27D_) without abrogation of receptor binding. The crystal structure of FL_L27D_ at 1.65 Å resolution revealed that the introduced point mutation led to shielding of the hydrophobic footprint of the dimerization interface in wild-type FL without affecting the conformation of the FLT3 binding site. Thus, FL_L27D_ can serve as a monomeric FL variant to further interrogate the assembly mechanism of extracellular complexes of FLT3 in physiology and disease.

## Abstract 2

This study reports the engineering and crystal structure of a monomeric variant of the hematopoietic cytokine FLT3 ligand that is able to bind to the cognate receptor. Such a tool can be used to interrogate the assembly mechanism of extracellular complexes of FLT3 and to further explore its therapeutic targeting.

## 1. Introduction   

**Figure 1:**
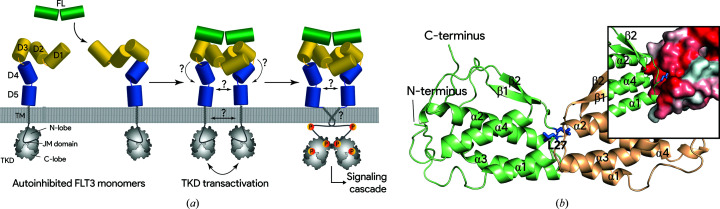
(*a*) FLT3 belongs to the class III receptor tyrosine kinase family, the members of which are characterized by a conserved modular build and activation mechanism. For all RTK-IIIs, cytokine ligands simultaneously bind to the membrane-distal domains (yellow; D1, D2 and/or D3) of two cognate receptors. Although this interaction has been shown to facilitate homotypic interactions between membrane-proximal domains (blue; D4 and/or D5) of almost all RTK-IIIs, this has not yet been demonstrated for the FL–FLT3 complex. The generation of such a ternary complex, possibly involving interactions of the transmembrane domains (TM), invokes a transphosphorylation of the inhibitory juxtamembrane (JM) domain, eventually resulting in fully activated kinase activity. (*b*) The dimeric interface of FL is centered around Leu27. A cartoon representation of FL (PDB entry 1ete; Savvides *et al.*, 2000 ▸) is shown with the constituting protomers colored green and sand yellow. Coloring according to the Eisenberg hydrophobicity scale (inset, surface representation; red is more hydrophobic) illustrates how Leu27 from each protomer (blue) is inserted into the hydrophobic interior of the other one.

Approximately 30% of newly diagnosed patients with acute myeloid leukemia (AML) harbor mutations in FMS-like tyrosine kinase receptor 3 (FLT3), which confer a poor disease prognosis (recently reviewed by Daver *et al.*, 2019 ▸). While the majority of such cases entail FLT3 with internal tandem duplications (ITDs) in the intracellular juxtamembrane region of the receptor (Nagel *et al.*, 2017 ▸; Tallman *et al.*, 2019 ▸; Daver *et al.*, 2019 ▸), somatic mutations in the extracellular and transmembrane domains of FLT3 have also been identified and at least one of them has been confirmed to be a driver mutation (Forbes *et al.*, 2008 ▸; Fröhling *et al.*, 2007 ▸). FLT3 is a transmembrane receptor that is expressed on the surface of early hematopoietic progenitor cells and dendritic cells. The receptor is a member of the class III tyrosine kinase receptors (RTK-IIIs), which include CSF-1R, KIT, PDGFRα and PDGFRβ, which are all characterized by a conserved modular architecture featuring an extracellular domain (ECD) comprising five Ig-like domains, a single membrane-spanning helix (TM) followed by a juxtamembrane (JM) region, and finally an intracellular tyrosine kinase domain (TKD) (Fig. 1 ▸ *a*; Lemmon & Schlessinger, 2010 ▸; Verstraete & Savvides, 2012 ▸).

Due to their highly similar build and the dimeric nature of their cognate cytokine ligands, RTK-IIIs are thought to be activated by similar mechanisms (Verstraete & Savvides, 2012 ▸). The binding of a dimeric cytokine to an RTK-III induces receptor dimerization that results in transactivation of the auto-inhibited tyrosine kinase domains and the activation of downstream signaling pathways (Fig. 1 ▸ *a*). While the intracellular activation mechanism of RTK-III is conserved in all RTK-IIIs, it has been shown that ligand binding to the membrane-distal domains takes place by homotypic receptor–receptor contacts that are mediated by the membrane-proximal Ig-like domains D4 and/or D5. Although such ligand-induced extracellular homotypic receptor interactions have been shown to be present in most RTK-IIIs (Elegheert *et al.*, 2011 ▸; Felix *et al.*, 2013 ▸, 2015 ▸; Yuzawa *et al.*, 2007 ▸; Chen *et al.*, 2015 ▸), they are absent in FLT3, as supported by the ‘open horseshoe’ structures of its complexes revealed via X-ray crystallography and negative-stain electron microscopy (Verstraete, Vandriessche *et al.*, 2011 ▸). Furthermore, the removal of two membrane-proximal domains of FLT3 resulted in only a moderate change in affinity for the ligand as determined by isothermal titration calorimetry, suggesting that these domains do not contribute significantly to the overall affinity of FLT3 for its cytokine (Verstraete, Vandriessche *et al.*, 2011 ▸).

As a consequence of the clear correlation between AML and mutations in FLT3, therapeutic targeting of FLT3 has been intensely pursued in industry and academia for over two decades (Badar *et al.*, 2015 ▸; Leick & Levis, 2017 ▸). With a few notable exceptions, most efforts have focused on the development of tyrosine kinase inhibitors addressing the intra­cellular domains of FLT3. With the advent of driver somatic mutations in the extracellular regions of FLT3, we hypothesized that more in-depth insights into the basic principles underlying FLT3 receptor activation could possibly reveal novel approaches to tackle constitutively activated oncogenic receptor variants. Indeed, despite having crystallographic models of FL and its complex with the ectodomains of FLT3 (Verstraete, Vandriessche *et al.*, 2011 ▸), the absence of structural insights into the possible inactive conformation of FLT3 and the conformational changes required to transition to an activated receptor–cytokine complex render our understanding of the extracellular complex principles incomplete.

To expand our molecular toolkit towards broadening the structural and mechanistic insights into FL–FLT3 assembly, we sought to engineer a monomeric variant of FL. The rationale behind such an endeavor was manifold. Firstly, a monomeric variant could be of use for the dissection of cytokine-mediated activation principles, as has been shown for CSF-1 (Elegheert *et al.*, 2012 ▸). Indeed, it has been suggested that Ig domain 1 of FLT3 could be involved in an inhibitory *cis* interaction with the membrane-proximal domains of the extracellular region (Verstraete & Savvides, 2012 ▸). Secondly, a monomeric ligand could be a starting point for the further engineering of tools to dissect receptor-activation principles in cellular assays, as previously illustrated for stem-cell factor variants (Ho *et al.*, 2017 ▸; Tilayov *et al.*, 2020 ▸) or as an *in vitro* binding probe that allows the discrimination of properly folded, binding-prone receptor species. Finally, we hypothesized that a non-activating, albeit receptor-binding-competent, variant of FL could lead to the stabilization of mechanistically relevant conformational states of FLT3.

## 2. Materials and methods   

### 2.1. Macromolecule production   

#### 2.1.1. Production of recombinant FLT3 ligands in *Escherichia coli*   

Recombinant wild-type FL (FL_WT_) and its Leu27Asp mutant (FL_L27D_) were produced according to previously published methods (Table 1 ▸; Verstraete *et al.*, 2009 ▸). Briefly, both proteins were expressed in an *E. coli* Rosetta-gami(DE3) bacterial strain (Novagen) as inclusion bodies. The harvested cell pellets were resuspended in lysis buffer (50 m*M* Tris pH 8.0, 100 m*M* NaCl, 1% Triton X-100, 1 m*M* EDTA) and lysed by sonication. Inclusion bodies were isolated, washed and solubilized in guanidine buffer (6 *M* guanidinium hydrochloride, 100 m*M* NaH_2_PO_4_, 10 m*M* Tris, 10 m*M* 2-mercaptoethanol pH 8.0) by gentle stirring at 40°C, followed by the strict application of previously published protocols (Verstraete *et al.*, 2009 ▸).

#### 2.1.2. Expression of recombinant proteins in mammalian cells and purification   

The cDNA sequence coding for human FLT3 domains 1–5 (FLT3_D1–D5_; residues Met1–Asp541) was obtained from Verstraete *et al.* (2009 ▸) and Verstraete, Remmerie *et al.* (2011 ▸). Constructs for transient mammalian expression of secreted proteins carrying a C-terminal thrombin-cleavable AviTag followed by a hexahistidine sequence were cloned in the pHLsec vector (Aricescu *et al.*, 2006 ▸). For the generation of stable cell lines, similar constructs were generated in the pcDNA4/TO vector (Thermo Fisher Scientific).

A monoclonal stable HEK293S *MGAT1* ^−/−^ TR^+^ cell line (Reeves *et al.*, 2002 ▸) was generated and grown to 90% confluence in the presence of 50 µg ml^−1^ zeocin (Verstraete, Remmerie *et al.*, 2011 ▸). To induce expression, the growth medium was replaced by serum-free medium supplemented with 2 µg ml^−1^ tetracycline and 3.6 m*M* valproic acid. After 4–5 days of transient or tetracycline-induced expression, the conditioned medium was harvested, cleared of cellular debris by centrifugation and filtered through a 22 µm cutoff bottle-top filter. Recombinant hexahistine-tagged proteins were captured from the conditioned medium by IMAC purification using a cOmplete His-tag purification column (Roche). After elution with 500 m*M* imidazole, the eluate was concentrated and further purified by size-exclusion chromatography using HiLoad 16/60 Superdex 75/200 columns (GE Healthcare) with HBS buffer (20 m*M* HEPES pH 7.4, 150 m*M* NaCl) as the running buffer. Protein purity was assessed by SDS–PAGE.

#### 2.1.3. Size-exclusion chromatography coupled to multi-angle laser light scattering (SEC-MALLS)   

Recombinant proteins and complexes thereof were concentrated to 1 mg ml^−1^ and injected onto a Superdex 200 Increase column (GE Healthcare) inline with an ultraviolet detector (Shimadzu), a multi-angle laser light scattering miniDAWN TREOS (Wyatt) and an Optilab T-rEX refractometer (Wyatt) at 25°C. HBS was used as the running buffer at a flow of 0.5 ml min^−1^. Data were analyzed using the *ASTRA*6 software (Wyatt). For the analysis of glycosylated protein species, conjugate analysis was performed using theoretical protein extinction coefficients and a d*n*/d*c* value of 0.16 for the glycan modifier (Bloch *et al.*, 2018 ▸).

### 2.2. Crystallization   

Recombinant FL_L27D_ was treated with 1 U µg^−1^ thrombin (New England Biolabs) overnight to remove the hexahistidine purification tag. Subsequently, thrombin and the cleaved peptide tag were separated from FL_L27D_ by size-exclusion chromatography. Sitting-drop vapor-diffusion crystallization trials were set up using a Mosquito crystallization robot (SPT Labtech) in nanolitre-scale Swissci 96-well triple-drop plates (Table 2 ▸). The protein plates were incubated at 293 K. Commercially available sitting-drop crystallization screens from Molecular Dimensions and Hampton Research were used to screen for conditions allowing crystal nucleation and growth. Seeding of crystallization conditions was performed using the Seed Bead Kit (Hampton Research) following the contemporary protocol.

### 2.3. Data collection and processing   

X-ray diffraction experiments were performed at 100 K on the PROXIMA-1 beamline at SOLEIL, Gif-sur-Yvette, France. Two wedges of diffraction data (1–90° and 120–180°) were indexed, integrated and scaled into a single data set using the *XDS* suite (Kabsch, 2010 ▸).

### 2.4. Structure solution and refinement   

The initial phases were obtained by maximum-likelihood molecular replacement in *Phaser* as implemented in the *CCP*4 package (McCoy *et al.*, 2007 ▸; Winn *et al.*, 2011 ▸) using a search model generated from the X-ray structure of FL (PDB entry 1ete; Savvides *et al.*, 2000 ▸). Structure building and refinement were performed iteratively using *Coot* (Emsley *et al.*, 2010 ▸), *Phenix* (Liebschner *et al.*, 2019 ▸) and *BUSTER* (Bricogne *et al.*, 2011 ▸). Final refinement was performed using *BUSTER* 2.10.3.

## 3. Results and discussion   

### 3.1. Engineering strategy to monomerize FL   

Mature wild-type FL belongs to the structural family of short-chain four-helical bundle cytokines and consequently exhibits a noncovalently linked homodimeric structure, in which the two protomers interact head to head (Savvides *et al.*, 2000 ▸). The availability of several crystallographic models of FL, both unbound (PDB entry 1ete) and in complex with its receptor (PDB entries 3qs7 and 3qs9), provided a structural basis for the development of a strategy to disrupt the dimeric interface of FL without introducing significant changes in the receptor-binding epitope (Savvides *et al.*, 2000 ▸; Verstraete, Vandriessche *et al.*, 2011 ▸). Following the strategy used to monomerize CSF-1 (Elegheert *et al.*, 2012 ▸), several constructs were generated with a tandem duplication of the dimer epitope (residues 18–30), some of which had one or multiple point mutations at sites playing a key role at the dimeric interface. However, despite extensive optimization of the purification protocols, we did not succeed in purifying a monomeric species that was stable in solution. Therefore, we resorted to a more targeted approach by introducing a single point mutation targeting Leu27 at the heart of the dimeric interface (Fig. 1 ▸ *b*). In each protomer, Leu27 is located at the tip of a loop formed by residues Leu26–Gln29, protruding into the hydrophobic interior of the four-helical bundle of the accompanying protomer. By mutating Leu27 to an aspartate, we hypothesized that the entropic penalty for burying a charged residue in the hydrophobic interior of the second protomer would be detrimental for any dimerization event to occur. Interestingly, previous work by Graddis *et al.* (1998 ▸) identified a Leu27-to-proline mutation in FL, based on random mutagenesis, that was deficient in dimerization at low protein concentrations.

### 3.2. FL_L27D_ is monomeric and engages in a 1:1 stoichiometric complex with FLT3   

**Figure 2:**
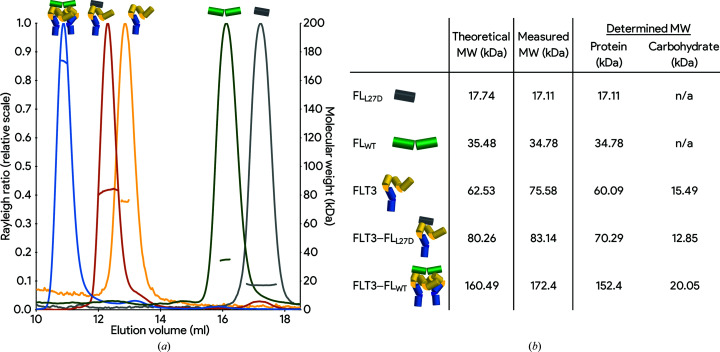
FL_L27D_ is a stable monomer capable of binding only one FLT3 molecule. (*a*) SEC-MALLS characterization of FL_WT_, FL_L27D_ and receptor complexes thereof. Elution profile monitored by the forward and right-angle laser detector (left axis) plotted against the SEC retention volume and overlaid with the measured molecular weight (right axis). FL_WT_ (green) is able to recruit two FLT3 molecules (yellow) into complex formation (blue), whereas FL_L27D_ (gray) binds FLT3 in an equimolar fashion (red). (*b*) Summary of the predicted molecular weights, based on the amino-acid sequence, and the MALLS-measured molecular weights. Further glycoprotein conjugate analysis of the latter allowed part of the mass to be attributed to the glycan content.

The expression of FL_L27D_ in *E. coli* followed by *in vitro* refolding of FL_L27D_ (Verstraete *et al.*, 2009 ▸) led to a stable and monodisperse protein that eluted in a size-exclusion chromatography (SEC) experiment as a protein with a substantially reduced hydrodynamic radius (*R*_hyd_) compared with wild-type FL (FL_WT_; Fig. 2 ▸, green and gray curves). Multi-angle laser light scattering (SEC-MALLS) analysis of these proteins during elution from SEC led to molecular-weight determinations of 35 and 17 kDa for FL_WT_ and FL_L27D_, respectively (Fig. 2 ▸ *b*). Importantly, no concentration-dependent dimerization could be detected even at concentrations as high as 95.83 µ*M* (1.7 mg ml^−1^). We concluded that these experimentally determined values are in excellent agreement with their theoretical molecular weights and confirmed that FL_L27D_ is indeed a monomeric species in solution.

To assess whether the monomer-inducing point mutation at position 27 affected the FLT3 binding site, which is localized close to the N-terminal region of FL (residues 6–13), we investigated its ability to form a complex with the extracellular region of recombinant human FLT3 comprising domains 1–5 (FLT3_D1–D5_; Verstraete, Remmerie *et al.*, 2011 ▸). The titration of a 20% molar excess of FL_L27D_ against FLT3_D1–D5_ and subsequent SEC-MALLS analysis resulted in a predominantly monodisperse species with an *R*_hyd_ exceeding that of both molecules alone (Fig. 2 ▸ *a*, red curve). With only an excess of FL_L27D_ detected, this shift indicates that despite its monomeric nature, FL_L27D_ was still able to recruit all available receptor molecules into complex formation. The molecular species has a molecular mass of 70 kDa as determined by SEC-MALLS, which is well below that of an FL-mediated receptor complex (152 kDa; Fig. 2 ▸ *b*) and therefore allowed us to infer that the apparent FL_L27D_–FLT3 complex consists of one molecule of FL_L27D_ and one molecule of FLT3.

### 3.3. Structural differences between FL_L27D_ and FL_WT_ are limited to the dimerization-interface region   

**Figure 3:**
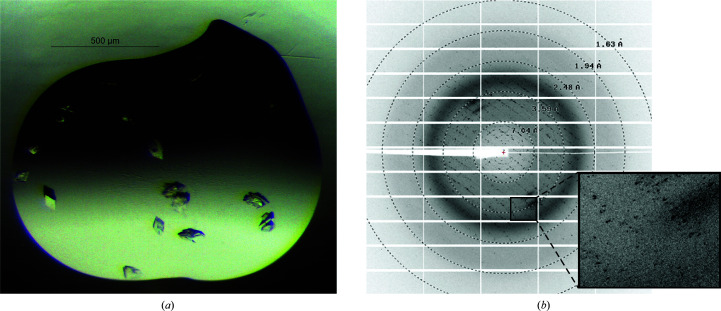
Representative crystal morphologies and corresponding X-ray diffraction from crystals of FL_L27D_. (*a*) Representative image of a crystallization drop containing crystals of FL_L27D_ displaying macroscopic crystal-growth pathologies. (*b*) Test X-ray diffraction image from the crystal that resulted in the data set used for obtaining the structure of the monomeric FL_L27D_ variant reported here. Resolution shells are displayed as circles. A close-up of the diffraction image (inset) reveals severe diffraction pathologies, including multiple lattices.

**Figure 4:**
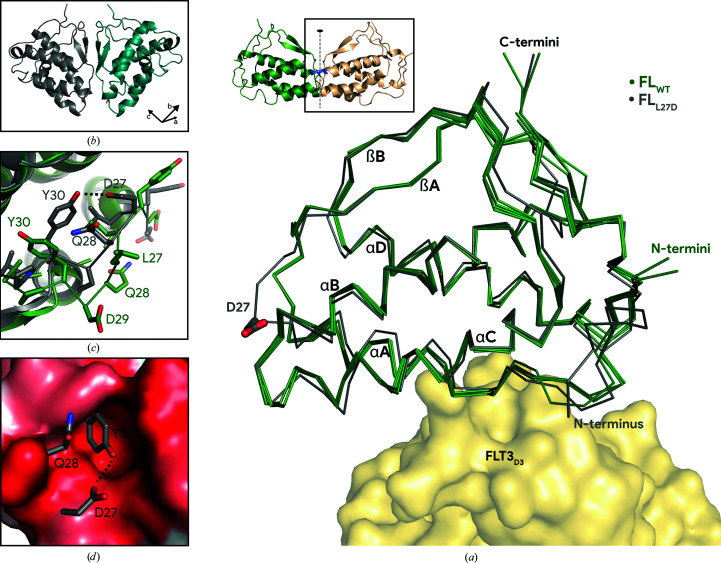
Structural differences between FL_L27D_ and FL_WT_ are limited to the dimerization-interface region. (*a*) Superimposition of FL_L27D_ (gray) and FL_WT_ (green). Crystallographic models of the ligands are shown in cartoon representation with indication of the twofold-symmetry axis (inset) or as ribbon diagrams (main panel); the side chain of Asp27 in FL_L27D_ is shown as sticks and FLT3 is shown in surface representation. With the exception of the αB–βA loop, the main chain of both FL_L27D_ molecules superimposes very well (average C^α^ r.m.s.d. of 0.85 Å) with the main chain of all four FL_WT_ copies (PDB entry 1ete). (*b*) The asymmetric unit of FL_L27D_ crystals features a top-to-top packing of molecules. This topology is distinct from the twofold-symmetry axis within one FL_WT_ molecule and supports the L27D mutation preventing dimerization even in the context of crystal packing. (*c*) Detail of the superimposed αB–βA loop of FL_L27D_ (gray) and FL_WT_ (green). Loop residues are shown as sticks. Hydrogen bonds are indicated by dashed lines. (*d*) Detail of the superimposed αB–βA loops of FL_L27D_ and FL_WT_, as viewed from the second FL_WT_ protomer. FL_WT_ is colored according the Eisenberg hydrophobicity scale (red is more hydrophobic); key residues of FL_L27D_ are shown as sticks. Hydrogen bonds are indicated by dashed lines.

To further validate that mutation of Leu27 to aspartate does not compromise the overall fold of the molecule, we pursued structural characterization of FL_L27D_ by X-ray crystallography. Initial crystallization trials resulted in the identification of multiple crystallization conditions across a wide pH range, all characterized by a high concentration (>1.8 *M*) of ammonium sulfate. Subsequent optimization of these initial hits led to crystals that diffracted synchrotron X-rays to high resolution, although all diffraction patterns showed signs of multiple crystal lattices (Fig. 3 ▸). Nevertheless, we were able to index at least one crystal into a single crystal lattice in space group *P*1 and used the obtained data to determine the crystal structure to 1.65 Å resolution (Tables 3 ▸ and 4 ▸, Fig. 4 ▸).

The obtained crystal structure of FL_L27D_ superimposes very well with a single protomer of FL_WT_ (Fig. 4 ▸ *a*). Indeed, not taking the αB–βA loop (residues 25–30) into account, the average root-mean-square deviation (r.m.s.d.) with FL_WT_ (PDB entry 1ete; Savvides *et al.*, 2000 ▸) is only 0.851 Å, indicating no large structural changes in the overall conformation of FL_L27D_. Given the observation that FL_L27D_ still binds FLT3, it comes as no surprise that the absence of structural deviation from FL_WT_ remains valid for residues 6–13, which are all key players in the largest interaction site of the FL–FLT3 epitope (Verstraete, Vandriessche *et al.*, 2011 ▸). Importantly, although the triclinic unit cell contains two copies of FL_L27D_ (Fig. 4 ▸ *b*) with apparent twofold rotational symmetry, the observed apparent symmetry axis is dramatically distinct in direction and context from the twofold-symmetry axis in dimeric FL_WT_ (Fig. 4 ▸ *a*, inset). Likewise, no combination of symmetry relations can reconstitute the head-to-head dimer resembling FL_WT_, despite the fact that the loop containing Asp27 is located near tightly packed crystal lattice contacts.

Given that the hydrophobic cavity that sheltered Leu27 of the accompanying FL_WT_ protomer would remain solvent-exposed after the L27D monomerization event, we wondered how FL_L27D_ would structurally compensate for this. When analyzing the conformational changes in the αB–βA loop (Fig. 4 ▸ *c*), we noticed that Asp27 is able to recruit Tyr30 via an intramolecular hydrogen bond, thus stabilizing the rotamer conformation of the latter such that it effectively shields the hydrophobic cavity that otherwise mediates dimeric FL_WT_ (Fig. 4 ▸ *d*). Thus, we have shown that FL can display structural plasticity in this region, which may open additional possibilities to engineer this part of FL for structure–function purposes.

## Supplementary Material

PDB reference: engineered monomeric FLT3 ligand, 7nbi

## Figures and Tables

**Table 1:** Macromolecule-production information

Forward primer	CGGCAGCCATATGACCCAGGACTGCTCCTTCC
Reverse primer	CGGATCCTTAGGGCTGACACTGCAGCTCCAGGC
Expression vector	pET-15b
Expression host	*E. coli* Rosetta-gami
Complete amino-acid sequence of the produced FL_L27D_ protein	MGSSHHHHHHSSGLVPRGSHMTQDCSFQHSPISSDFAVKIRELSDYLDQDYPVTVASNLQDEELCGGLWRLVLAQRWMERLKTVAGSKMQGLLERVNTEIHFVTKCAFQPPPSCLRFVQTNISRLLQETSEQLVALKPWITRQNFSRCLELQCQP
Amino-acid sequence of the thrombin-digested FL_L27D_ protein	GSHMTQDCSFQHSPISSDFAVKIRELSDYLDQDYPVTVASNLQDEELCGGLWRLVLAQRWMERLKTVAGSKMQGLLERVNTEIHFVTKCAFQPPPSCLRFVQTNISRLLQETSEQLVALKPWITRQNFSRCLELQCQP

**Table 2:** Crystallization

Method	Microseeding in combination with vapor diffusion
Plate type	Swissci 96-well 3-drop plates
Temperature (K)	293
Protein concentration (mg ml^−1^)	22
Buffer composition of protein solution	20 m*M* HEPES, 150 m*M* NaCl pH 7.4
Composition of reservoir solution	2.0 *M* ammonium sulfate, 0.1 *M* HEPES pH 5.0
Volume and ratio of drop	1:1
Volume of reservoir (µl)	45
Cryoprotectant	None

**Table 3:** Data collection and processing Values in parentheses are for the outer shell.

Diffraction source	PROXIMA-1, SOLEIL, France
Wavelength (Å)	0.97625
Temperature (K)	100
Detector	PILATUS 6M
Crystal-to-detector distance (mm)	321.8
Rotation range per image (°)	0.1
Total rotation range (°)	180
Exposure time per image (s)	0.2
Space group	*P*1
*a*, *b*, *c* (Å)	28.30, 43.49, 46.36
α, β, γ (°)	82.82, 85.41, 85.10
Mosaicity (°)	0.105
Resolution range (Å)	18.42–1.65 (1.709–1.650)
Total No. of reflections	70278 (4053)
No. of unique reflections	24967 (1712)
Completeness (%)	94.9 (89.2)
Multiplicity	2.81 (2.37)
〈*I*/σ(*I*)〉	10.6 (2.33)
Overall *B* factor from Wilson plot (Å^2^)	16.95
*R*_meas_ (%)	7.7 (56.7)
CC_1/2_ (%)	99.6 (72.8)

**Table 4:** Structure refinement Values in parentheses are for the outer shell.

Resolution range (Å)	18.42–1.65 (1.709–1.65)
No. of reflections, working set	24910 (2403)
No. of reflections, test set	1246 (120)
Final *R*_cryst_	0.1643
Final *R*_free_	0.2026
No. of non-H atoms	
Total	2436
Protein	2193
Ligand	25
Water	218
No. of protein residues	269
R.m.s.d., bond lengths (Å)	0.017
R.m.s.d., angles (°)	1.49
Ramachandran favored (%)	98.11
Ramachandran allowed (%)	1.89
Ramachandran outliers (%)	0.00
Rotamer outliers (%)	0.00
Clashscore	8.42
Average *B* factors (Å^2^)	
Overall	21.57
Protein	20.48
Ligands	50.19
Solvent	29.2
No. of TLS groups	1
